# A new mode of clinical failure of porous tantalum rod

**DOI:** 10.4103/0019-5413.69322

**Published:** 2010

**Authors:** Kwang-Jun Oh, Dilbans Singh Pandher

**Affiliations:** Departmetnt of Orthopaedics, Konkuk University Hospital, Seoul, South Korea; 1 Oxford Super-specialty and Trauma Hospital, Jalandhar, India; 2 SGL Charitable Hospital, Subhanpur, Kapurthala

**Keywords:** Tantalum rod, osteonecrosis, femur head

## Abstract

The area of osteonecrosis of the head of femur affected by the disease process varies from a small localized lesion to a global lesion. Without specific treatment 80% of the clinically diagnosed cases will progress, and most will eventually require arthroplasty. Therefore the goal is to diagnose and treat the condition in the earliest stage. A number of surgical procedures have been described to retard or prevent progression of the disease and to preserve the femoral head. An implant made of porous tantalum has been developed to function as a structural graft to provide mechanical support to the subchondral plate of the necrotic femoral head, and possibly allow bone growth into the avascular region. Porous tantalum implant failure with associated radiological progression of the disease is reported in the literature; however, there is no report of clinical failure of the implant without radiological progression of the disease. We report a case of clinical failure of porous tantalum implant, seven months after surgery without any radiological progression of the disease, and with histopathological evidence of new bone formation around the porous tantalum implant. The patient was succesfully treated by total hip arthroplasty.

## INTRODUCTION

The area of osteonecrosis of the head of femur affected by the disease process varies from a small localized lesion to a global lesion. Without specific treatment 80% of the clinically diagnosed cases will progress, and most will eventually require arthroplasty.^1^ Therefore the goal is to diagnose and treat the condition in the earliest stage. A number of surgical procedures have been described to retard or prevent progression of the disease and to preserve the femoral head. Free vascularized fibular grafting has been reported to provide satisfactory pain relief and functional improvement.^2^–^6^ However, a major limitations of the free vascularized fibular grafting is high rate of complications associated with the procedure and lengthy surgery.^3^,^7^–^9^ To overcome this limitation, an implant made of porous tantalum was recently developed to function as a structural graft to provide mechanical support to the subchondral plate of the necrotic femoral head. The rationale for the use of the tantalum is that the high porosity of the material, its fully interconnected pores, the osteoconductive micro texture on the tantalum struts, and an elastic modulus that is similar to that of the cancellous bone will provide mechanical support and possibly allow bone growth into the avascular femur head.^10^–^12^ Few studies have reported early failure of porous tantalum implant with radiological progression of the disease.^13^–^16^ We report a case of clinical failure of porous tantalum implant, seven months after surgery without any radiological progression of the disease, and with histopathological evidence of new bone formation around the porous tantalum implant.

## CASE REPORT

**Figure 1:**
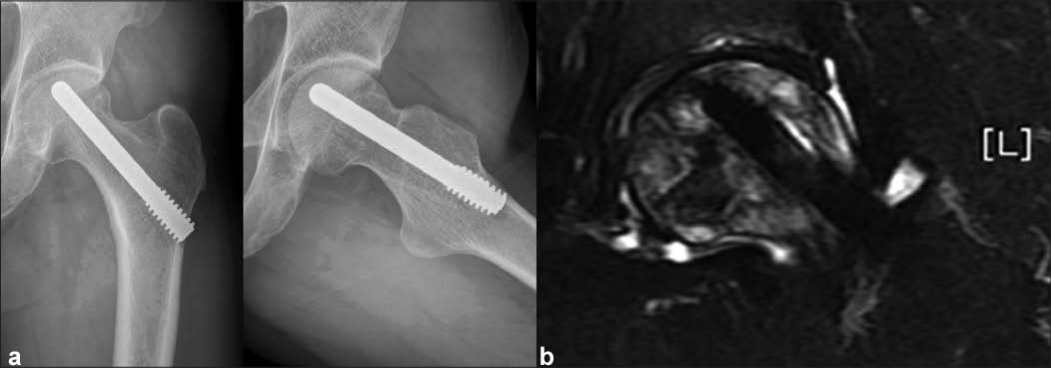
(a) Postoperative radiograph anteroposterior and lateral views showing a well placed implant with no signs of subchondral collapse or depression in the articular surface. (b) Follow-up MRI scan showing porous tantalum rod in the necrotic area, with reactive marrow signal changes around the tip of the implant, without any evidence of femoral head collapse

**Figure 2:**
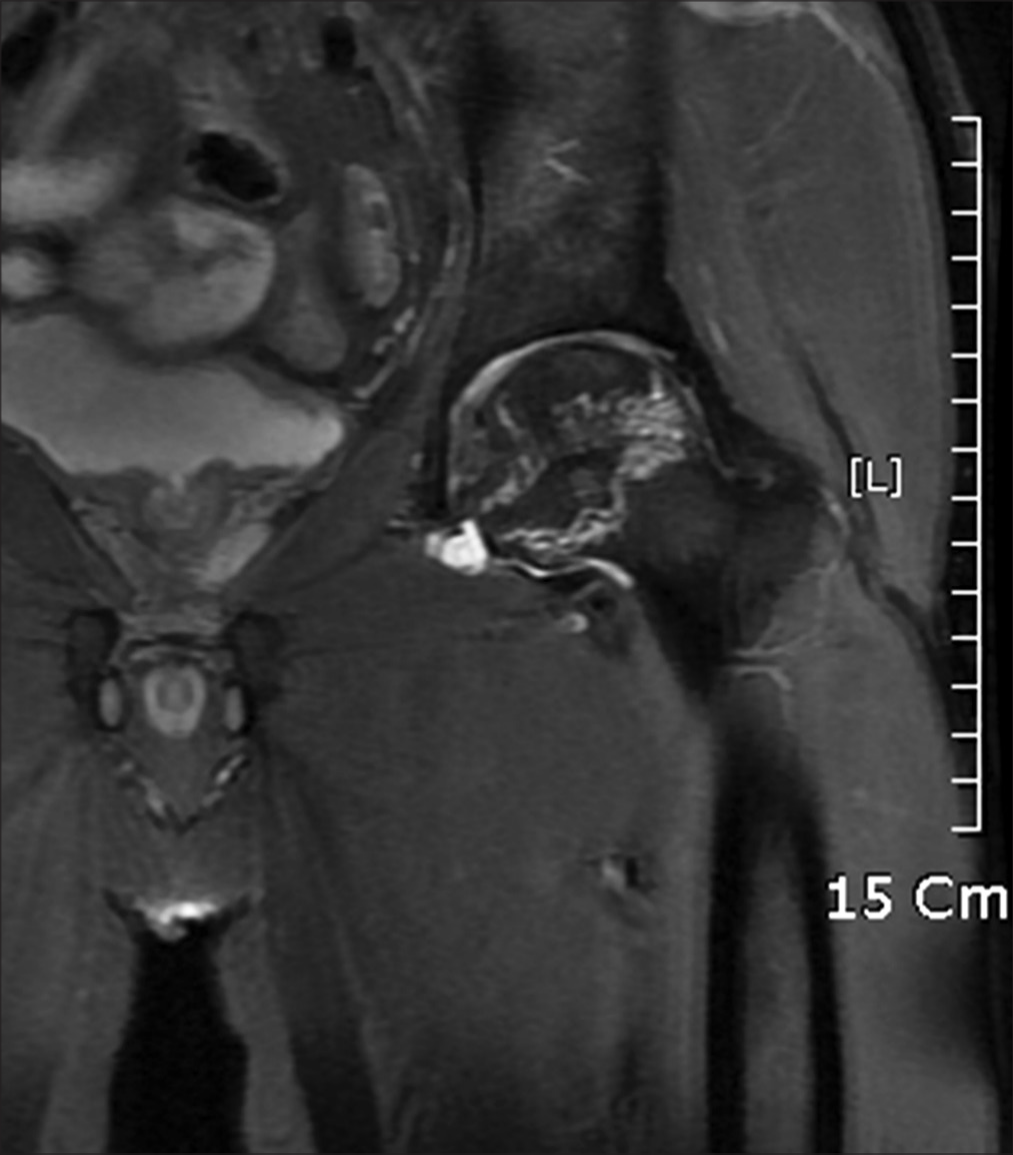
MRI scan showing large osteonecrotic lesion involving more than 80% geographical area of articular surface of the head of femur with MR crescent sign

A 42-year-old male patient presented to out patient department (OPD) with a complaint of severe pain in right hip joint. After routine clinical and radiological examination, he was diagnosed to be suffering from advanced osteonecrosis of the right femoral head. Total hip arthroplasty (THA) was planned for the right hip. At the same time he had a mild pain in left hip joint. Radiographs of the left hip joint were normal. Taking into consideration the patient complaint and osteonecrosis of the right femur head, magnetic resonance imaging (MRI) scan was performed for the left hip joint to rule out early osteonecrotic changes as a cause of pain. MRI revealed large osteonecrotic lesion involving more than 80% geographical area of articular surface with MR crescent sign [Figure 1]. There was no apparent collapse of the subchondral bone. The Hospital for Special Surgery (HSS) score of left hip was 68 points at the time of surgery. Core decompression and porous tantalum rod insertion was done for the left hip joint. Postoperative period was uneventful. Patient was advised non-weight bearing mobilization with use of crutches for six weeks, followed by gradual increase to full weight bearing as tolerated. He had complete relief from pain and full range of motion for seven months after surgery. After that he complained of mild pain of gradual onset in the left hip and groin region. Radiographic examination showed a well placed implant with no signs of subchondral collapse or depression in the articular surface [Figure 2]. Patient was prescribed analgesic medication and advised to follow-up after one month. At the next follow-up, patient complained of worsening of pain which hampered his daily living activities. MRI scan of the left hip joint was performed to evaluate the status of osteonecrosis. The scan showed a well located metal implant (porous tantalum rod) in the necrotic area, with reactive marrow signal changes around the tip of the implant, without any evidence of femoral head collapse [Figure 2]. The reactive marrow signal was reported to be, probably revascularization signal change or reactive edema due to implant insertion. Patient was reassured about the clinical and radiological status of the disease process and prescribed analgesic medication. However, patient attended OPD after three weeks with excruciating pain and insisted on total hip arthroplasty (THA) for the left hip joint. The HSS score worsened to 78.6 points on last follow-up. Considering this case as a clinical failure of tantalum rod, THA was performed on the left hip joint. The femur head with tantalum rod in situ was sent for histopathological examination (HPE). Patient had complete relief from pain after THA on the left hip joint. The HSS score the case improved from 79 preoperatively to 96 points postoperatively.

**Figure 3:**
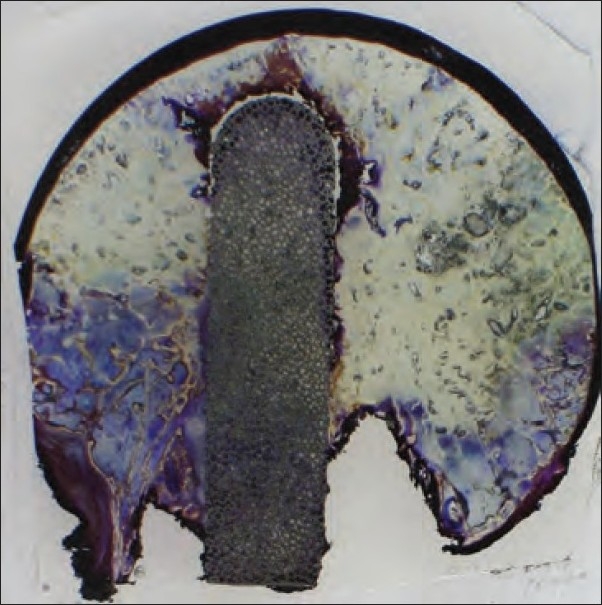
Histopathological slide on gross examination showing well placed implant in the center of necrotic area, with apparent new bone formation around the tip and the margins of the implant

On gross examination, the implant was well placed in the center of necrotic area [Figure 3]. There was a small gap between the implant and the adjacent bone at the distal interface with apparent new bone formation around the tip and the margins of the implant.

**Figure 4:**
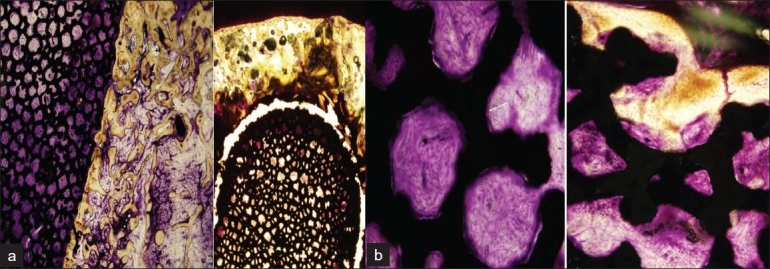
(a) 12.5× image showing well formed bony trabeculae in contact with implant surface without gap (Lt). Cancellous bone around tip is not new bone formation (Rt). (b) 100× image showing implant pores with active proliferation of young fibroblasts in vascular rich stroma and dense celullar rim lining the surface of implant material (Rt). (The cellular rim is supposed to be a possible osteoblastic proliferation that could not be technically evaluated in specimen.) Several foci of ingrowing new bone into porous implant sprouting from interface zone were evident (left, arrow)

The HPE [Figure 4a] at 12× magnification revealed well formed bony trabeculae in contact with the implant surface without gap, but the cancellous bone around the tip was not new bone formation [Figure 4a]. The implant revealed active proliferation of young fibroblasts in vascular rich stroma and dense cellular rim lining the surface of the implant material [Figure 4b]. The cellular rim was supposed to be a possible osteoblastic proliferation that could not be technically evaluated in the specimen. On 100× magnification, several foci of new bone sprouting from the interface zone into the porous implant were evident [Figure 4b]. Dead loose connective tissue existed between the implant and irregularly disarrayed cancellous marrow bone at the tip of the implant.

These findings could be compared with failure cases in our study, which had associated radiological progression. The pores of the failed implants had dead marrow tissue with fat necrosis and infiltration of chronic inflammatory cells.

## DISCUSSION

Porous tantalum has demonstrated bone ingrowth and rapid fixation in animal models^17^,^18^ and in human explant case reports.^19^–^20^ It has similar flexural rigidity to the human fibula, thereby providing mechanical support to the subchondral plate while limiting stress shielding.^10^,^17^ The operative technique is simple, free from risks and complications as compared to vascularized fibular grafting. The surgical procedure accomplishes two functions: (i) by drilling and reaming up to the joint cartilage, it is possible to decompress the femoral epiphysis and remove necrotic tissue; (ii) with the implant directed towards the osteonecrotic site, it is possible to provide a mechanical support to the joint surface and hopefully initiate the repair process in the osteonecrotic area.

In our case, the porous tantalum rod had provided effective mechanical support to the joint surface as there was no collapse of the articular surface on MRI or HPE of the retrieved femoral head with implant in situ.

A retrieval study has reported small shards of bone stacked up on the rounded tip of the implant in nine of the fifteen cases,^21^ which were confirmed on transmitted microscopy to be necrotic shards of bone at the tip of the implant. The shards showed no evidence of remodeling and had the appearance of necrotic reamed bone rather than femoral head osteonecrosis. The same study found no bone ingrowth in the porous tantalum in two cases and minimal bone ingrowth in the rest, with an average of only 1.9%, much less than the mean density (26.2%) of adjacent femoral head cancellous bone.^21^ It reported no evidence of new bone formation or vascular invasion in the osteonecrotic portion of the femoral head. In the present case, bone ingrowth was noticed on porous tantalum which was continuous and surrounded the implant. The tip of the implant was covered by cancellous bone, which on gross examination appeared to be new bone growing into osteonecrotic area of head, but on histopathological examination no new bone growth was found.

It seems that this continuous shell of new bony ingrowth might have nullified the effect of core decompression, by blocking the porous tantalum. Thick mantle of cancellous bone around the tip, which appeared to be reactive new bone formation on MRI might have added to the blocking effect. Though the porous tantalum rod provided good structural support to the articular cartilage and helped maintain the integrity of the articular surface, the new bone formation blocked the porous rod completely, probably leading to gradual increase in the intramedullary pressure and patient started experiencing pain. The pain was severe enough to quote this case as clinical failure and plan THA for the affected hip.

The current case suggests a new mode of clinical failure of porous tantalum rod, by which the new bony ingrowth leads to blockage of the core decompression effect of the porous tantalum, leading to arise in the intramedullary pressure and reappearance of the clinical symptoms of osteonecrosis. The iatrogenic part seems to be the cancellous bone around the tip of the tantalum rod, which is not new bone, but may be cancellous bone from neck that was pushed along the tip of the implant during insertion. In the suggested scenario, steps should be taken to avoid this iatrogenic factor in the clinical failure of the porous tantalum rod.
